# Case report: Diagnostic value of [99mTc]Tc-sestamibi SPECT/CT in a very small renal mass: a histopathology-matched case

**DOI:** 10.3389/fmed.2026.2003763

**Published:** 2026-10-28

**Authors:** Luca Urso, Gamze Beydagi, Alessia Cimadamore, Corrado Cittanti, Nicola Pegoraro, Domenico Albano, Mirco Bartolomei, Alessandro Crestani, Gianluca Giannarini

**Affiliations:** 1 Department of Translational Medicine, University of Ferrara, Ferrara, Italy; 2 Nuclear Medicine Unit, Onco-Hematology Department, University Hospital of Ferrara, Ferrara, Italy; 3 Department of Nuclear Medicine, University of Health and Sciences Turkey, Istanbul Training and Research Hospital, Istanbul, Türkiye; 4 Department of Medicine, Institute of Pathological Anatomy, University of Udine, Udine, Italy; 5 Radiology Unit, Department of Diagnostic Imaging and Laboratory Medicine, Arcispedale Sant'Anna, Ferrara, Italy; 6 Nuclear Medicine, University of Brescia, Brescia, Italy; 7 Urology Unit, Santa Maria Della Misericordia University Hospital, Azienda Sanitaria Universitaria Friuli Centrale (ASUFC) – University of Udine, Udine, Italy; 8 Department of Medicine (DMED), University of Udine, Udine, Italy

**Keywords:** CAIX imaging, molecular imaging, renal mass, renal tumor, risk stratification, sestamibi, SPECT

## Abstract

A 12-mm enhancing renal mass was incidentally detected in a 61-year-old man during CT urography. To refine characterization, [^99m^Tc]Tc-sestamibi SPECT/CT was performed, showing radiotracer uptake comparable to the surrounding cortex (L/K ratio 0.95) despite the sub-centimetric size, suggestive of an oncocytic or otherwise indolent lesion. Given patient-specific factors and contralateral renal impairment, partial nephrectomy was undertaken, revealing a renal oncocytoma with typical histopathological and immunohistochemical features. This case highlights that, although technical limitations exist for lesions <2 cm, [^99m^Tc]Tc-sestamibi SPECT/CT may still provide interpretable molecular information in selected very small renal masses, especially when integrated with CAIX-targeted radiotracers – such as girentuximab – whose complementary biological profile enhances noninvasive renal mass assessment. The concordance between imaging and histology supports further investigation into standardized acquisition and interpretation criteria to optimize noninvasive assessment and guide management of small renal masses.

## Introduction

The characterization of incidentally detected small renal masses (SRM) poses several challenges. Conventional modalities such as computed tomography (CT) and magnetic resonance imaging (MRI) often have limited reliability in distinguishing benign from malignant lesions when their size is small (i.e., below 4 cm). As a result, several benign entities - including oncocytoma, and hybrid oncocytic chromophobe tumors (HOCT) - may appear radiologically indistinguishable from malignant renal cell carcinoma (RCC) subtypes, in particular clear cell RCC (ccRCC) (1).

Percutaneous renal mass biopsy (RMB) remains the most definitive method for establishing the nature of a renal mass (2). However, RMB is an invasive procedure with inherent risks and may yield non-diagnostic samples, delaying the diagnostic process (3). Moreover, technical expertise is essential, and certain anatomical locations - particularly anterior and medial or deeply endophytic lesions - can further complicate the procedure.

[^99m^Tc]Tc-sestamibi Single-Photon Emission Computed Tomography (SPECT/CT) has emerged as a promising adjunct for the noninvasive characterization of indeterminate renal masses (4). The biological rationale underlying this technique is [^99m^Tc]Tc-sestamibi's tropism for mitochondria-rich oncocytic tumors (5). Conversely, most malignant renal neoplasms, particularly ccRCC, exhibit reduced tracer retention due to active efflux mediated by multidrug-resistance pumps, a mechanism well know in cancer chemoresistance (5, 6). Published series have reported higher tracer avidity and higher lesion-to-kidney (L/K) ratios in benign oncocytic lesions than in malignant renal masses (7, 8). However, lesions smaller than 2 cm remain challenging for SPECT/CT characterization, due to partial volume effects related to low-energy gamma emissions and the limited spatial resolution of current imaging systems (9).

## Case presentation

A 61-year-old man, with no significant comorbidities, was referred for CT urography to evaluate a known left-sided hydronephrosis, in the context of recurrent nephrolithiasis, marked functional impairment of the residual parenchyma, and multiple recurrent infectious episodes. CT urography confirmed a left kidney of markedly reduced size with diffuse cortical thinning, at times barely discernible, and calyceal corticalization with mild dilatation. The renal pelvis showed a mildly irregular contour without significant dilatation. In the delayed excretory phase, opacification of the calyceal system and minimal contrast passage into the pelvis were observed, with absent opacification of the left ureter.

The right kidney appeared normal in size and morphology, with preserved parenchymal thickness, and contained two tiny lower-pole calyceal calculi measuring up to 2 mm each. At the posterior aspect of the mid portion of the right kidney, CT incidentally identified a 12-mm solid, slightly exophytic cortical mass producing a focal cortical bulge and demonstrating marked arterial-phase enhancement with mild venous washout (Figure 1). The mass was absent on previous CT scans and was considered suspicious for a neoplastic process of unclear biological behavior (benign vs. malignant).

**Figure 1:**
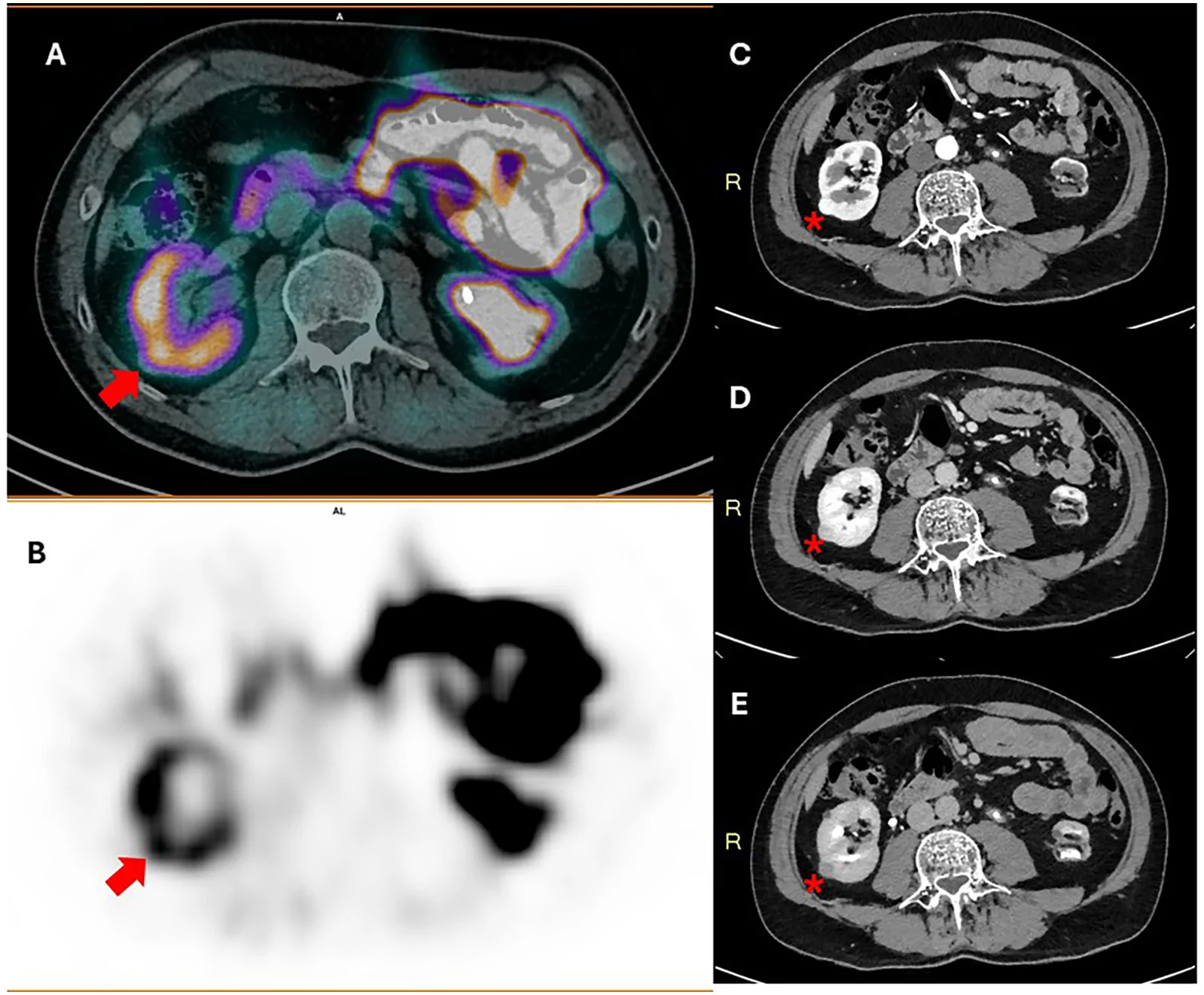
Multimodal imaging of a right renal small mass, lately diagnosed as oncocytoma. **(A)** Fused axial [^99m^Tc]Tc-sestamibi SPECT/CT showing radiotracer uptake in the renal cortex; the red arrows indicate the cortical nodule with uptake, comparable to surrounding parenchyma, as can be seen also from SPECT images **(B) (C–E)** Axial contrast-enhanced CT images of the right kidney in cortico-medullary **(C)**, nephrographic **(D)**, and excretory **(E)** phases; the lesion (red asterisk) demonstrates marked arterial-phase enhancement with mild venous washout.

To further characterize the lesion noninvasively, [^99m^Tc]Tc-sestamibi renal scintigraphy was performed. The examination was conducted after intravenous administration of 740 MBq (20mCi) of [^99m^Tc]Tc-sestamibi, with early abdominal planar imaging at 5 min post-injection, delayed planar imaging at 85 min, and upper-abdominal SPECT/CT acquisition at 90 min (Figure 1).

At scintigraphic images, the known cortical nodule in the mid-to-lower pole of the right kidney demonstrated radiotracer uptake essentially equivalent to that of the surrounding renal parenchyma. Semiquantitative analysis yielded a lesion-to-kidney (L/K) ratio of 0.95. The scintigraphic findings were therefore consistent with a renal nodule of likely benign nature, supporting radiological follow-up or, if clinically indicated, histological confirmation.

Despite the very small size of the lesion and the [^99m^Tc]Tc-sestamibi findings suggesting an oncocytic or otherwise biologically indolent phenotype, management options were discussed with the patient, including active surveillance and histological characterization by RMB. Given his relatively young age, the newly developed and enhancing nature of the lesion, and the severely impaired function of the contralateral kidney, the patient preferred definitive treatment. Partial nephrectomy was therefore performed with the aim of obtaining definitive histological diagnosis and oncological control while maximizing preservation of the solitary functioning kidney.

Histopathological examination of the resected specimen revealed a well-circumscribed, unencapsulated tumor composed of medium-sized polygonal cells with abundant granular eosinophilic cytoplasm and round, uniform nuclei. Immunohistochemical staining demonstrated strong and diffuse positivity for CD117, with scattered CK7-positive cells, confirming the diagnosis of renal oncocytoma. (Figure 2).

**Figure 2:**
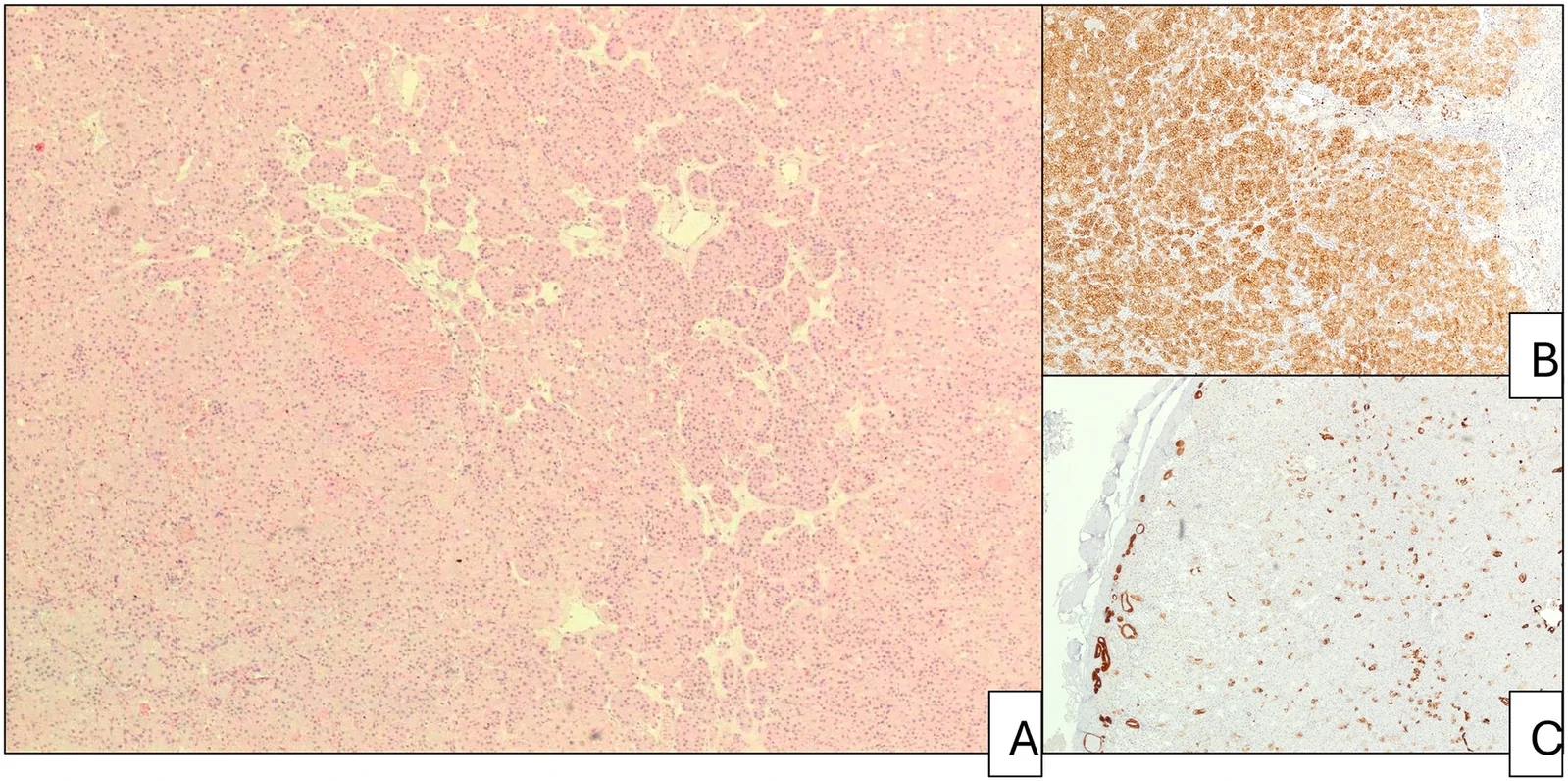
Renal oncocytoma showing a trabecular and nested growth pattern, composed of eosinophilic cells with uniform, round nuclei (**A**, H&E, ×4). Strong and diffuse CD117 immunoreactivity is observed **(B)** CK7 immunostaining highlights scattered tumor cells **(C).**

## Discussion

The literature has described the high diagnostic accuracy of [^99m^Tc]Tc-sestamibi SPECT/CT for the characterization of renal masses, undetermined at conventional imaging. A recent metanalysis reports a sensitivity of 89% and a specificity of 98% for the non-invasive discrimination of renal oncocytomas and HOCT vs. ccRCC and papillary RCC (pRCC) (1). Nevertheless, most masses imaged in currently available literature had a diameter larger than 3 cm (1). This case is notable because the lesion measured only 12 mm, well below the 2-cm threshold generally considered suitable for reliable SPECT/CT characterization (9). Nevertheless, the preoperative imaging interpretation was fully concordant with the final histopathological diagnosis of a benign lesion (renal oncocytoma). Although this single observation cannot establish diagnostic accuracy below the conventional 2-cm threshold, it suggests that interpretable [^99m^Tc]Tc-sestamibi findings may occasionally be obtained even in renal masses approaching 1 cm, provided that accurate SPECT/CT coregistration and appropriate reconstruction techniques are available. Due to the intrinsically high background activity of normal renal parenchyma, it is plausible that lesions smaller than the conventional size threshold for SPECT imaging could still be assessed with [^99m^Tc]Tc-sestamibi SPECT/CT, as they may manifest as focal defects within the cortical uptake. Nevertheless, in lesions of this size, interpretation remains particularly challenging because uptake comparable to the surrounding renal cortex may render the mass visually inconspicuous, while partial-volume effects and spill-in from adjacent parenchyma may affect semiquantitative measurements.

Our case supports further investigation into the technical feasibility and diagnostic performance of [^99m^Tc]Tc-sestamibi SPECT/CT0020in renal masses smaller than 2 cm. Given its broad availability and relatively low cost (10), [^99m^Tc]Tc-sestamibi SPECT/CT may serve as a useful triage test for indeterminate renal masses, contributing - together with anatomical imaging, clinical characteristics, biopsy feasibility, and patient preference - to the selection of patients for active surveillance, histological characterization, or treatment. Furthermore, the use of [^99m^Tc]Tc-sestamibi SPECT/CT along with carbonic anhydrase IX (CAIX) targeting radiotracers (i.e., [^89^Zr]Zr-girentuximab) is under investigation and could further improve a patient-tailored approach. Choi and colleagues (11) recently proposed a framework integrating molecular imaging into the structured Kidney Imaging Reporting and Data System (KI-RADS), for non-invasive renal mass characterization and management (12). The score is based on the complementary nature of [^99m^Tc]Tc-sestamibi SPECT/CT and CAIX-targeting radioagents, with the latter identifying with very high sensitivity and specificity ccRCC as “hot” masses (13). Therefore, a situation with a positive [^99m^Tc]Tc-sestamibi SPECT/CT and a negative [^89^Zr]Zr-girentuximab PET/CT could address to a safe surveillance of a renal mass (KI-RADS score =1). The main limitation that can be currently identified in this approach is the limited availability of CAIX-targeted radioagents, together with the scheduling and radiation protection drawbacks of [^89^Zr]-labelled agents, like [^89^Zr]Zr-girentuximab, that was used in the phase 3 ZIRCON trial (14). Some new small molecules targeting CAIX are under investigation as PET probes, i.e., [^68^Ga]Ga-DPI-4452, could represent a solution to the issues currently hindering the widespread of these imaging agents (15).

Another relevant unmet need is the availability of a recognized structured reporting for [^99m^Tc]Tc-sestamibi SPECT/CT. In a previous study from our group (4), we proposed a three-tier visual scoring system for sestamibi SPECT/CT interpretation, designed to standardize the qualitative assessment of radiotracer uptake in renal masses. This framework - ranging from absent to clearly increased uptake - was conceived to support lesion stratification and to complement semi-quantitative parameters such as the lesion-to-kidney ratio. The present case aligns with that conceptual model, as the lesion exhibited uptake comparable to the surrounding cortex (grade 3) and achieved a moderately-high L/K ratio, consistent with an oncocytic profile. The use of this structured scoring tier could improve the generalizability of the results of [^99m^Tc]Tc-sestamibi SPECT/CT and aid the integration of a molecular imaging KI-RADS in clinical practice and further refine the simple visual assessment currently suggested by current guidelines (16).

In conclusion, our case demonstrates histopathologically concordant [^99m^Tc]Tc-sestamibi uptake in a 12-mm renal oncocytoma, despite the lesion being below the size generally considered optimal for SPECT/CT characterization. The finding supports the technical feasibility of molecular characterization in selected very small renal masses but should not be interpreted as evidence of diagnostic reliability in this size range. [^99m^Tc]Tc-sestamibi avidity primarily suggests an oncocytic or biologically indolent phenotype but its results should be integrated with anatomic imaging, biopsy feasibility, renal functional reserve, and patient preferences. Prospective studies using standardized acquisition, interpretation, and semiquantitative criteria are required to determine whether [^99m^Tc]Tc-sestamibi SPECT/CT can meaningfully reduce unnecessary biopsy or surgery in lesions smaller than 2 cm, either as a standalone modality or in combination with CAIX-targeted radiotracers.

## Data Availability

The original contributions presented in the study are included in the article/Supplementary Material, further inquiries can be directed to the corresponding author.
